# Inhaled platelet vesicle-decoyed biomimetic nanoparticles attenuate inflammatory lung injury

**DOI:** 10.3389/fphar.2022.1050224

**Published:** 2022-11-29

**Authors:** Hua Jin, Renxing Luo, Jianing Li, Hongxia Zhao, Suidong Ouyang, Yinlian Yao, Dongyan Chen, Zijie Ling, Weicong Zhu, Meijun Chen, Xianping Liao, Jiang Pi, Gonghua Huang

**Affiliations:** ^1^ Guangdong Provincial Key Laboratory of Medical Molecular Diagnostics, The First Dongguan Affiliated Hospital, Guangdong Medical University, Dongguan, China; ^2^ School of Pharmacy, Guangdong Medical University, Dongguan, China; ^3^ School of Medical Technology, Guangdong Medical University, Dongguan, China; ^4^ School of Biomedical and Pharmaceutical Science, Guangdong University of Technology, Guangzhou, China

**Keywords:** acute lung injury, biomimetic nanoparticle, Chinese herbal medicine (CHM), platelet membrane coating, macrophage polarization (MP), histone lactylation

## Abstract

Acute lung injury (ALI) is an inflammatory response which causes serious damages to alveolar epithelia and vasculature, and it still remains high lethality and mortality with no effective treatment. Based on the inflammatory homing of platelets and cell membrane cloaking nanotechnology, in this study we developed a biomimetic anti-inflammation nanoparticle delivery system for ALI treatment. PM@Cur-RV NPs were designed by combining the poly (lactic-co-glycolic acid) nanoparticles (NPs) coated with platelet membrane vesicles (PM) for the purpose of highly targeting delivery of curcumin (Cur) and resveratrol (RV) to inflammatory lungs. PM@Cur-RV NPs showed good biocompatibility and biosafety both *in vitro* and *in vivo*. Accumulation of NPs into lung tract was observed after inhaled NPs. Remarkably, the inhalation of PM@Cur-RV NPs effectively inhibited lung vascular injury evidenced by the decreased lung vascular permeability, and the reduced proinflammatory cytokine burden in an ALI mouse model. The analysis of infiltrated macrophages in the lungs showed that the Cur-RV-modulated macrophage polarized towards M2 phenotype and the decreased histone lactylation might contribute to their anti-inflammation effects. Together, this work highlights the potential of inhalation of biomimetic nanoparticle delivery of curcumin and resveratrol for the treatment of pulmonary diseases.

## Introduction

Acute lung injury (ALI) or acute respiratory distress syndrome (ARDS) is characterized by respiratory distress, refractory hypoxemia, and non-cardiogenic pulmonary edema, with significantly high morbidity and mortality in critically ill patients (Zhang et al., 2021a). COVID-19 is associated with acute respiratory distress and cytokine release syndrome. In the later stage of disease, some COVID-19 patients may develop into ALI/ARDS or even multiple organ failure. Despite an improvement in the lung protective ventilation and a fluid conservative strategy, the mortality rate remains as high as 40% (Prasanna et al., 2021). In clinics, ALI/ARDS treatment is mainly relying on hormone drugs, such as glucocorticoids, antibiotics and supportive care (Fernando et al., 2021). Although all of these drugs demonstrate significantly anti-inflammatory effects, their off-targeting in the body determines their seriously systemic immunosuppressive side effects (Oray et al., 2016). Therefore, the findings of new targets and the development of targeting strategies are of great significance for controlling clinical pneumonia from mild to severe, reducing severe mortality, shortening hospitalization time, and improving the life quality of patients during treatment.

Macrophages are one of the most important immune cells in the body to control the inflammatory responses. Abundance of studies has shown that macrophages are key mediators in the pathogenesis of ALI/ARDS (Huang et al., 2018a), which demonstrates that regulation of macrophage polarization might be an important strategy to improve the prognosis. Under different local environmental stimuli, macrophages can be divided into two distinct polarization states: classically activated phenotype (M1), and the alternatively activated phenotype (M2) (Chen et al., 2020). Substantial evidences have shown that M1 is associated with pro-inflammatory responses, while M2 makes a great contribution to anti-inflammatory reactions (Sica and Mantovani, 2012; Arora et al., 2018). This demonstrates that employment of novel drugs or strategy to regulate macrophage towards M2 phenotype might be an effective treatment for ALI/ARDS.

Curcumin (Cur), a diarylheptanoid, which is produced from plants of the curcuma longa species, has been shown excellent inhibition effects in inflammation, metabolic syndrome, pain, various of cancers, etc (Hewlings and Kalman, 2017). In recent years, Cur has been reported to inhibit the inflammation through the modulation of macrophage autophagy (Shakeri et al., 2019). Although most of these pharmacological activities of curcumin demonstrate to play roles at the cellular level, it can’t achieve expective improvements in clinical therapeutics due to its poor absorption, rapid metabolism, and rapid elimination (Ghalandarlaki et al., 2014).

Resveratrol (3, 5, 4' -trihydroxystilbene, RV) is a natural phytoalexin which is widely presents in grapes, peanuts, berries and especially Chinese herbal medicine Polygonum cuspidatu. Resveratrol is attracting increasing attentions in many fields, such as medicine, pharmacy, etc, because of its good anti-proliferative, anti-oxidative and anti-inflammatory activity (Zhang et al., 2021b). It has been found that RV could attenuate lung vascular hyperpermeability during ventilator-induced lung injury. However, its poor solubility in aqueous solvents greatly restricts its efficiency, bioavailability and application in clinics.

Therefore, the targeted delivery and enhancing the bioavailability of anti-inflammatory agents to the local inflammation sites represent a promising strategy for disease treatment. Recently, cell membrane coating-based biomimetic nanodrug delivery system has attracted increasing attentions in the field of nanomedicine (Fang et al., 2018). Cell membrane coated nanoparticles (NPs) are developed combining a NP core coated with membrane or membrane vesicles derived from natural cells such as erythrocytes, endothelial cells, cancer cells, stem cells, platelets or bacterial cells. This kind of biomimetic engineered device has proven to possess the functions of proteins from source cell membrane and drugs loaded in nanoparticles. The targeting ability of these biomimetic nanoparticles is often mediated by proteins that are expressed on the source cells, and this facilitates the NPs with the ability to specifically adhere to various disease substrates. In the past decade, various of cell membranes from erythrocytes to cancer cells have been coated onto the surface of nanoparticles to obtain nano-formulations with enhanced functionality that can be custom-tailored to specific applications (Park et al., 2021). Among these, nanoparticles coated with the membrane derived from platelets were develop to target damaged vasculature or inflammation sites in the lungs because platelets have the intrinsic affinity to the site of inflammation (Ma et al., 2020).

In this work, to improve the bioavailability and facilitate delivery of Cur and RV to the lung inflammations sites, poly (lactic-co-glycolic acid) (PLGA) nanoparticles were synthesized to co-encapsulate Cur and RV, and platelet membrane were isolated from healthy mouse blood and coated onto the Cur-RV NPs to form the inflammation targeting PM@Cur-RV NPs. *In vitro*, PM@Cur-RV NPs showed good dispersity, excellent biocompatibility and biosafety. *In vivo* studies demonstrated that PM@Cur-RV NPs could target and accumulate into the inflammatory lungs. In LPS-induced ALI mouse model, the PM@Cur-RV NPs demonstrated enhanced inhibiting effects on lungs vascular permeability and impressive improvement in anti-inflammation of lungs compared with other formulations of Cur and RV (i.g., Free Cur-RV and Cur-RV NPs). Interestingly, the PM@Cur-RV NPs could induce macrophages towards M2 polarization in pulmonary alveoli. Collectively, our study demonstrates that this kind of biomimetic nano-platform might be an efficient strategy to deliver drugs for treating infectious diseases, such as pneumonia.

## Methods and materrials

### Preparation of Cur and RV-coloaded PLGA nanoparticles

The Cur-RV NPs were synthesized using emulsification and evaporation method as previously described (Jin et al., 2017) with a little modification. Briefly, 20 mg of Curcumin ((Sigma, United States), 20 mg of resveratrol (Shanghai yuanye Bio-Technology Co., Ltd) and 160 mg PLGA-PEG (lactide:glycolide 50:50, sigma, United States) were co-dissolved in 5 ml of dichloromethane (Tianjin Damao Chemical Reagent Factory, China) as oil phase (O), 20 ml of PVA (1%, w/w, from sigma, United States) was as external water phase (W). Firstly, the O phase was ultrasonic for 40 s on ice bath to form the first emulsification. Then, the first emulsification was dropped in W phase and ultrasonicated for another 40 s to form the second emulsification. After that, the second emulsification was added to 100 ml water and stirred for 6 h for organic regent evaporation and nanoparticle hardening. Finally, the nanoparticles were harvested by centrifuging at 12,000 rpm for 20 min and washed 3 times using ultrapure water. The harvested NPs were lyophilized for 48 h for storage in powdered form.

### Isolation of platelets

Fresh whole blood samples were drawn from healthy mice. Platelets from whole blood were isolated through gradient centrifugation [15]. Briefly, 0.5 ml whole blood (from five mice) was centrifuged at 200 g for 10 min and isolated the supernatant as platelet-rich plasma (PRP). Then, the PRP was centrifuged at 1800 g for 20 min, remove the supernatant and the precipitation was platelets. Wash it with PBS two times before using.

### Preparation of platelet membrane-coated Cur and RV-coloaded NPs

Platelets were suspended in deionized water with protease inhibitor, frozen at −80°C, thawed at room temperature for use. The process of frozen-thawing was repeated for three times. A BCA protein assay kit (Solarbio, China) was used to determine the protein concentration of isolated PM. Finally, the mixture was extruded *via* a Hand Extruder to obtain uniform particles.

### Characterization of NPs

Size distribution of NPs was measured using a Zetasizer Nano ZS. NPs were visualized by scanning electron microscopy (Philips Co., Holland). The encapsulation of Cur and RV was detected using an ultraviolet and visible spectrophotometer (UV 6000). To measure the drug (Cur and RV) loading rate of Cur-RV NPs, 10 mg lyophilized nanoparticles were dissolved in 1 ml of methanol, and then the amount of Cur or RV in solution was determined by High Pressure Liquid Chromatography (HPLC). HPLC detection was performed using a C18 column (5 μm, 250 mm × 4.6 mm). Whereas the mobile phase, consisting of methanol and 0.1% acetic acid (88:12) (v/v), was maintained at a flow rate of 1.0 ml/min. The ultraviolet detector wavelength was 218 nm (for Cur) and 210 nm (for RV) and the injection volume was 20 µl. The loading of Cur and RV was calculated the concentration of Cur and RV based on the standard curve.

### SDS-PAGE analysis of retention proteins from cell membranes and NPs

Retained membrane proteins were examined by SDS-PAGE. Stem cell membrane protein samples and PM@Cur-RV NPs were mixed with loading buffer (Beyotime, China) and denatured at 95°C. 30 μg of protein samples was separated by 10% SDS-PAGE gel, and then the gel was stained with Coomassie Blue for 2 h and decolored overnight for imaging.

### Hemolysis assay

To determine the *in vivo* biosafety of the compounds, hemolysis of red blood cells treated with the designated compounds was determined. Erythrocytes were originated from normal healthy C57BL/6J mice through washing the anticoagulant whole blood with PBS at 3,500 rpm for 5 min. Then, a 4% red blood cell suspension (v/v, in PBS) was blended with different compounds, including nanoparticles, MLT, and different formulations of DOX and ICG. The erythrocyte sample lysed using pure water was used as positive control, and erythrocyte sample diluted using PBS was used as negative control group. After incubating with 100ug/mL of compounds at 37°C for 4 h, all the samples were centrifuged at 3,500 rpm, 4°C, for 5 min to collect the supernatant and measured its absorbance value at 550 nm by a microplate reader. Also, the red blood cells were added into 12-well plate and imaged using a Living cell imaging system (EVOSFL Auto, Invitrogen, United States).

### Cell viability assay

Cell Counting Kit-8 assay was used to detect cell viability according to the manufacturer’s instruction. Primary cultures of Human umbilical vein endothelial cell (HUVECs) and human bronchial epithelial cell line (HBE cells) were cultured at 37°C in a humidified atmosphere of 5% CO_2_ and 95% air. HUVECs or HBE cells were seeded into 96-well plates at a density of 10^4^ cells/well in 100 µl of RPMI medium with 10% FBS and incubated for 24 h. Then, the medium was replaced with 100 µl of fresh culture medium containing LPS (1ug/mL) plus either PBS with 5% of DMSO (vehicle) or different formulations of Cur and RV for another 24 h. Cell viability was then estimated by CCK-8 assay (Beyotime institute of biotechnology, China)) according to the manufacturer’s protocol.

### Cellular uptake

The green fluorescence of Cur was as the fluorescent marker of the different formulations of Cur and RV. Human bronchial epithelial cells (HBE) were bought from ATCC (Shanghai, China). The cells were cultured in DMEM (Gibco, United States) with 10% fetal bovine serum (Gibco) containing 100 μg/ml streptomycin and 100 IU/ml penicillin at 5% CO_2_ and 37°C. The cells were cultured with free Cur-RV, Cur-RV NPs and PM@Cur-RV NPs for 4 h, separately, and then washed twice with PBS to remove unbound nanoparticles and added into PBS. The cells were imaged using a Live Cell Imaging System (EVOSFL Auto, Invitrogen, United States).

### Animal study

8–12-week-age female C57/6J mice were purchased from SPF biotechnology co., LTD., Beijing, China. Experimental procedures using mice in this work were reviewed and approved by the ethical review board of Guangdong Medical University, and all the experiments were performed in accordance with relevant guidelines and regulations of Animal Ethics Committee of Guangdong province, China.

### Mouse model of acute lung injury

Lipopolysaccharide (LPS, *E.coli* 0111:B4, L2630) was bought from Sigma (United States). To induce acute lung injury, LPS was dissolved in PBS and administered by intraperitoneal injection (i.p.) to mice at 5 mg/kg body weight in 150 µl PBS.

### *In vivo* imaging of NPs in mice

LPS-induced C57BL/6J mouse sepsis model were intranasally administered with 50 μl of differently modified nanoparticles. Indocyanine green (ICG) was loaded into the NPs as fluorescence marker. For semiquantitative analysis, the image and fluorescence intensities of mice were collected 2 h after intranasal administration of NPs and determined using the Kodak Multi Mode Imaging System.

### Assessment of lung vascular permeability

The Evans blue-conjugated albumin (EBA) extravasation assay was used to assess pulmonary vascular permeability (Zhao et al., 2014). Briefly, EBA (20 mg/kg) was injected retro-orbitally at 30 min before sacrifice and lung collection following perfusion free of blood with PBS. The extravasated EBA in lung homogenates was expressed as μg of EBA per mg of lung. Briefly, lung homogenates were incubated with two volumes of formalin for 18 h at 60°C, centrifuged at 5,000 g for 30 min, and the optical density (OD) of the supernatant was determined spectrophotometrically at 620 nm.

Total protein levels in bronchiolar alveolar lavage fluid (BALF) were measured *via* bicinchoninacid-assay (BCA) according to the manufacturer’s instructions (Pierce BCA Protein Assay, Thermo Scientific, United States).

#### RT-PCR analysis of cytokines in lung tissues

To determine the expression of mRNA in lungs, lungs from asthmatic mice were lysed with a tissue homogenizer in TRIzol (Invitrogen). Total RNA was extracted by TRIzol according to the manufacturer’s instructions. Real-time PCR analysis was performed with primers using Power SYBR Green Master Mix from Life Technologies. All gene expression results (mRNA abundances) were expressed as arbitrary units relative to the abundance of GADPH mRNA. The fold change was calculated through the 2^−ΔΔCT^ method. The primers used were as follows: The primers used were as follows: TNF-α, forward primer: CCC​TCA​CAC​TCA​GAT​CAT​CTT​CT, reverse primer: GCT​ACG​ACG​TGG​GCT​ACA​G; IL-6, forward primer: TAG​TCC​TTC​CTA​CCC​CAA​TTT​CC, reverse primer: TTG​GTC​CTT​AGC​CAC​TCC​TTC; iNOS, forward primer: TAC​TGA​GAC​AGG​GAA​GTC​TGA​A, reverse primer: AGT​AGT​TGC​TCC​TCT​TCC​AAG​GT; ICAM1, forward primer: GTG​ATG​CTC​AGG​TAT​CCA​TCC​A, reverse primer: CAC​AGT​TCT​CAA​AGC​ACA​GCG.

### Histology

Lung tissue and other organs (liver, spleen, kidney) were fixed and processed for H and E staining. Briefly, lung tissues were fixed by 5 min instillation of 10% PBS-buffered formalin through trachea catheterization at a transpulmonary pressure of 15 cm H_2_O, and then overnight at 4°C with agitation. The other organs (liver, spleen, kidney) were isolated and fixed using 10% of formalin at 4°C with agitation for 48 h. After paraffin processing, the tissues were cut into 5 μm thick and stained with H&E for histological analysis. Inflammation scores were determined as: grade 0, no inflammation; grade 1, occasional cuffing with inflammatory cells; and grades 2, 3, and 4 indicated that most bronchi or vessels were surrounded by a thin 1–2 cell layer, a moderate 3–5 cell layer, or a thick (>5) cell layer of inflammatory cells, respectively (Xing et al., 2019).

### Assay of aspartate aminotransferase and alanine transam

The levels of AST and ALT were determined using Elisa kit (Nanjing Jiancheng Bioengineering Institute, Nanjing, China)) according to the manufacturer’s instructions. Take 100 mg of liver tissue and add 900 µl of saline, and then homogenized, centrifugated, and the supernatant was kept for test.

### Lung mononuclear cells isolation

Lung mononuclear cells were prepared as previously described (Han et al., 2022). Briefly, lung tissues were sliced into small pieces and incubated at 37°C for 45 min with collagenase IV (1 mg/ml; Life Technologies) in RPMI-1640 medium (HyClone) supplemented with 5% fetal bovine serum (FBS; HyClone), and cells were isolated by gradient centrifugation over 38% Percoll (GE Healthcare Life Sciences). After erythrocyte lysis with ACK lysis buffer (Gibco), the cells were harvested for analyses.

### Flow cytometric analysis for macrophage polarization in lung

For surface staining, lung mononuclear cells were labeled with FITC-mouse CD11b (48-0112-82, eBioscience)PE/cyanine7-anti-mouse CD11c (117317; Biolegend), APC-anti-mouse F4/80 (123115; Biolegend) and FITC-anti-mouse CD206 (141703; Biolegend) and the matching control isotype IgG (MCA421; AbD Serotec) in FACS buffer (PBS with 2% FBS) for 30 min at 4°C. M1 macrophages were identified as F4/80+CD11c+CD206– and M2 macrophages were identified as F4/80 + CD11c–CD206+ cells. Flow cytometry data were acquired on BD LSRFortessa X-20 and analyzed using FlowJo software (Tree Star).

### Western blot

Whole proteins extracted from lung and protein concentrations were determined using BCA protein assay. Samples containing 40 µg of protein were fractionated by SDS-polyacrylamide gel electrophoresis (PAGE) and electro-transferred to a PVDF membrane. The blocked membranes were then immunoblotted with primary antibodies Anti-L-Lactyl Lysine Rabbit mAb (1:1,000, PTMBIO Inc., China) and anti-β-actin (1:1,000, Cell Signaling Technology Inc. United States). The proteins were visualized using enhanced chemiluminescence by secondary antibody. WB bands were scanned and analyzed for optical density using ImageJ software.

### Statistical analysis

Pairwise comparisons were analyzed using non-parametric unpaired Student’s t-tests for equal variance. Multiple comparisons were assessed using ANOVA with Bonferroni post-tests. *p* values of less than 0.05 were considered statistically significant. Values are mean ± standard deviation (SD).

## Results

### Characterization of PM@Cur-RV NPs

**FIGURE 1:**
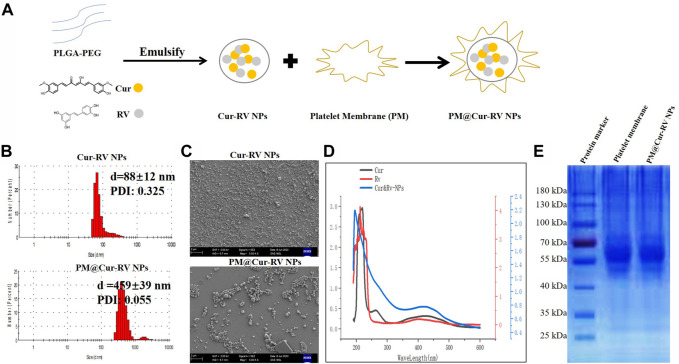
Characterization of Cur and RV-coloaded PLGA nanoparticles. **(A)** Scheme illustrates the preparation of Cur-RV NPs and PM@Cur-RV NPs using emulsification method. **(B)** Size distribution of Cur-RV NPs and PM@Cur-RV NPs, respectively. **(C)** Representative morphology photos of Cur-RV NPs and PM@Cur-RV NPs imaged by scanning electron microscope (1bar = 2 μm). **(D)** UV-vis spectrum of free Cur, RV and Cur-RV NPs indicated that Cur and RV were both successfully encapsulated in Cur-RV NPs, which shown by the similar absorption peaks at around 210 nm. **(E)** SDS-PAGE protein analysis of platelet membrane (PM), and PM@Cur-RV NPs (the samples were determined at same protein concentrations).

A classical emulsification-evaporation method was used to prepare the PLGA NPs. During the synthesis process, the compounds Cur and RV were added into the oil phase of the PLGA NPs. The harvested PM@Cur-RV NPs were mixed with platelet membrane and extruded through a polycarbonate porous membrane to form PM@Cur-RV NPs (shown in Figure 1A). This as-synthesized biomimetic nano-system was supposed to achieve inflammation targeting, and facilitate the delivery of drugs (Cur and RV) into the lungs in LPS-induced sepsis mice.

Nano Particle Analyzer SZ-100 (Horiba Scientific) and scanning electron microscope (SEM) were employed to characterize the size and morphology of Cur-RV NPs and PM@Cur-RV NPs. As shown in Figure 1A, the sizes of Cur-RV NPs were around 100 nm, and the sizes were remarkably increased into 450 nm with the membrane coating. The SEM image (Figure 1C) indicated that these NPs presented a spherical and uniform morphology, and PM@Cur-RV NPs demonstrated a better dispersion.

The UV-vis spectrum of the NPs was measured to determine the loading of Cur and RV in NPs. As shown in Figure 1D, the absorption spectrum of Cur or RV exhibited the characteristic absorption peaks at 210 nm. The absorption spectrum of the PM@Cur-RV NPs was closely consistent with that of free Cur or RV, indicating a successful encapsulation of Cur and RV into the PM@Cur-RV NPs. The loading capacity of Cur and RV was ∼70% and ∼75% in Cur-RV NPs and PM@Cur-RV NPs, which was calculated based on the standard curve of Cur and RV at their UV absorption peak of 425 nm and 208 nm, separately.

For further confirmation of the PM coating onto the Cur-RV NPs, the protein ingredients of PM and PM@Cur-RV NPs were analyzed by SDS-gel electrophoresis (Huang and Zhao, 2012). As shown in Figure 1E, PM@Cur-RV NPs contained characteristic proteins preserved by stem cell membrane (indicated by red circle). Collectively, these results indicate that the proteins of PM are successfully coated or conjugated on the surface of the Cur-RV NPs.

### Biocompatibility and biosafety of the NPs

**FIGURE 2:**
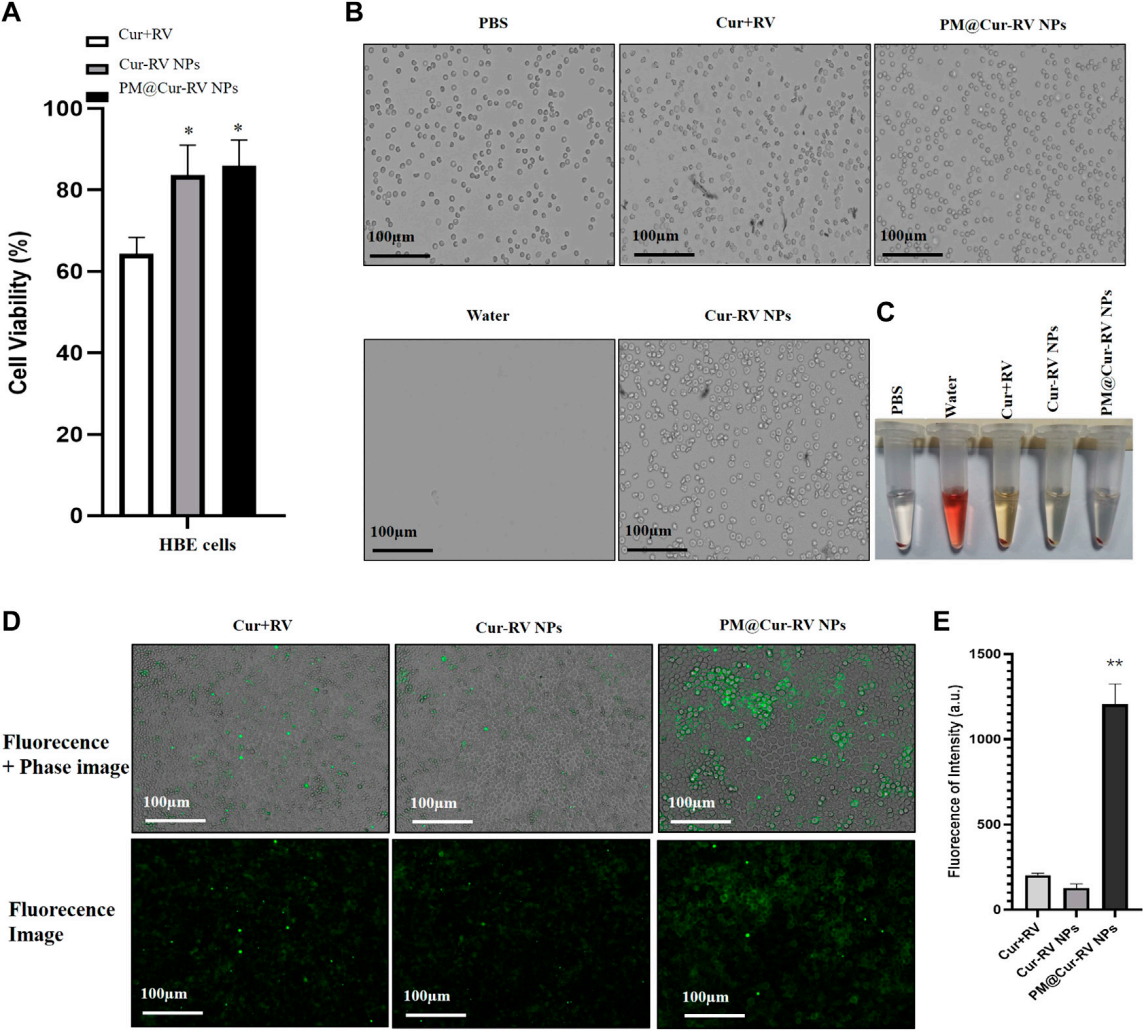
Biocompatibility and biosafety of different formulations of Cur and RV. **(A)** Cell viability of human bronchial epithelial cell line (HBE cells) treated with different formulations of Cur and RV at 100 μg/ml for 24 h, which implied that low dose of Cur and RV will not cause cytotoxicity to cells. **(B)** The images of hemolysis assay of free Cur and RV, Cur-RV NPs and PM@Cur-RV NPs, separately. **(C)** The morphology of erythrocytes co-cultured with free Cur and RV, Cur-RV NPs and PM@Cur-RV NPs, separately. **(D)** The typical images of HBE cell uptake of free Cur-RV, Cur-RV-NPs and PM@Cur-RV NPs (green fluorescence resulted from Cur). **(E)** The fluorescence intensity in HBE cells cocultured with different formulations of Cur-RV.

Giving that the biocompatibility and biosafety of nanoparticle-based systems have been known as the most important factors for their clinical application, the effects of free Cur and/or Cur-RV NPs were performed by using alveolar epithelial cell line (HBE cells) and endothelial cell line (HUVECs). As shown in Figure 2A, the same concentration of Cur-RV NPs and PM@ Cur-RV NPs did not induce significant cytotoxicity of macrophages, and only slightly inhibited the cell viability of HBE cells and HUVECs. Notably, 100 μg/ml of free Cur and RV had significant inhibiting effects on the viability of HBE cells and HUVECs, implying that the high dose of free Cur and RV could induce toxic effects on lung epithelia and endothelia. This result indicates that nano-formulation of compounds can significantly decrease their toxicity and increase their biosafety.

To further confirm the biosafety of Cur and RV, we evaluated their hemolytic properties using healthy mouse red blood cells. The erythrocytes lysed by water were used as the hemolysis positive group and the erythrocytes dispersed in PBS buffer were used as the normal control group. The free compounds, Cur-RV NPs and PM@Cur-RV NPs were used at the same concentration of 100 μg/ml (calculated based on the Cur and RV loading into NPs). As shown in Figures 2B,C, the free compounds showed slightly hemolysis. Interestingly, after being formulated into the NPs, the erythrocytes treated with Nar-NPs or CM@Nar-NPs did not show hemolysis. In addition, the shapes of erythrocytes under different treatments were also imaged under a light microscope. The same numbers of erythrocytes were added into 12-well plate, and 100 μg/ml different formulations of compounds were added and cultured for 30 min in 37°C, with pure water or PBS as hemolysis positive or normal control group, separately. As shown in Figure 2C, the water-lysis erythrocytes were almost completely destructed with a few of cell fragments, while the PBS-treated group showed normal, healthy erythrocytes with intact double concave disc shapes. In free compound group, the erythrocytes showed slightly decreased in the number and deformed in the shapes, while erythrocytes co-cultured with Cur-RV NPs and PM@Cur-RV NPs showed the intact membrane and healthy shapes. Collectively, these data indicate that nanoparticle-based platform can significantly improve the biocompatibility and biosafety of free Cur and RV.

### *In vivo* targeting of PM@Cur-RV NPs

**FIGURE 3:**
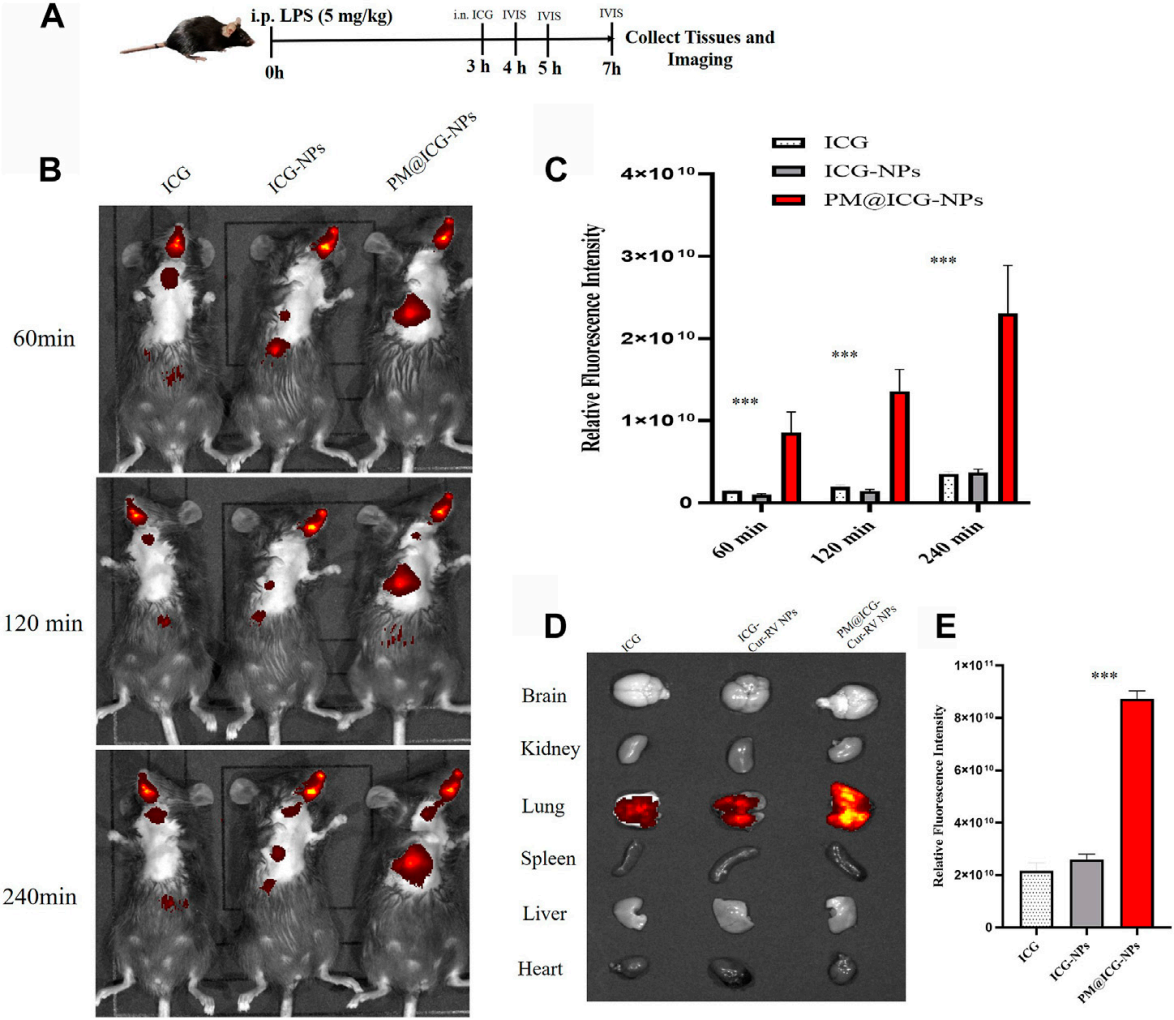
*In vivo* targeting of different modified NPs. Indocyanine green (ICG) were co-loaded into the NPs as a fluorescent marker of nanoparticles. **(A)** Scheme of time axis shows the IVIS imaging of ALI mice: the mice were intraperitoneal (i.p.) administrated with LPS (5 mg/kg) to induce ALI model and at 3 h post LPS challenging, the mice were intranasally administrated with different formulations of ICG. **(B)** Representative IVIS imaging of free ICG, ICG-NPs or PM@ICG-NPs distributed in sepsis mice at 1, 2, and 4 h after intranasal administration of ICG. **(C)** Relative fluorescence intensity of ICG in Figure B, which was measured with Living Image 4.5 software. Data are expressed as mean ± SD (n = 4). *p < 0.05, **p < 0.01 *versus* ICG group. **(D)** Typical fluorescence imaging of main organs originated from ALI mice after intranasal of Nar-NPs with or without PM modification for 4 h. **(E)** Relative fluorescence intensity of ICG in Figure D, calculating with Living Image 4.5 software.

**FIGURE 4:**
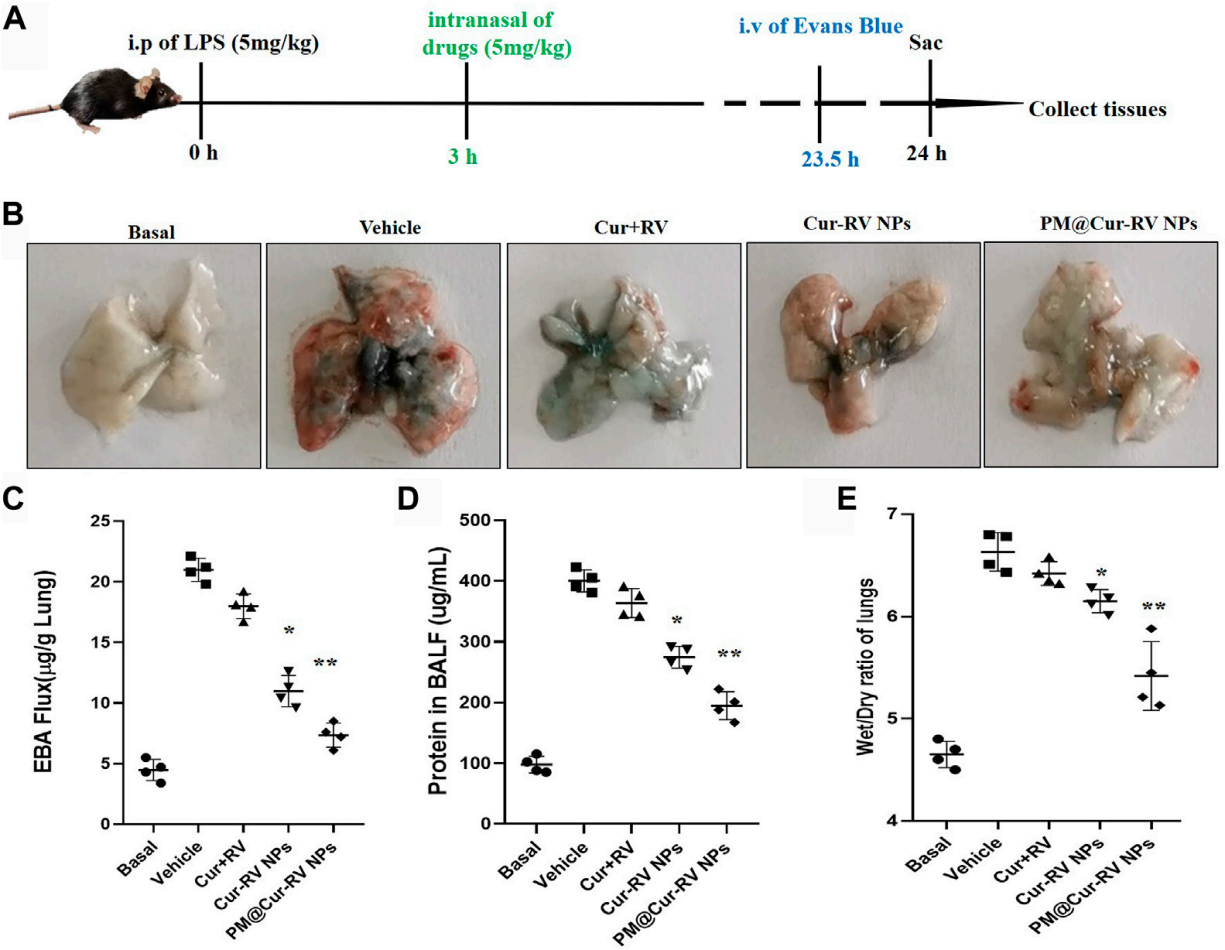
Cur-RV NPs, especially PM@Cur-RV NPs significantly inhibit lung vascular permeability in LPS-challenged mice. **(A)** Time-axis of lung vascular permeability determined by EBA (Evans blue-conjugated albumin) extravasation assay. At 3 h post-LPS challenge (5 mg/kg, i. p.), drugs (5 mg/kg, i. n.) were administered to mice. The same volume of PBS (5% of DMSO were included) was administrated to a group of mice as controls. Lungs were collected at 24 h post-LPS challenging for quantitative tests. **(B)** Representative photos demonstrated that curcumin-NPs could significantly inhibited lung vascular injury comparing with that of curcumin group. At 24 h post-LPS challenge, mouse lungs 30 min after i. v. Of EBA were perfused to remove blood and imaged. **(C)** The amounts of Evans Blue (EB) fluxed in lungs were assayed to evaluate the permeability of lung vessels. **(D)** The quantitative analysis of protein level in broncholveolr lvge fluid (BALF) of mice treated with different formulations of Cur and RV. **(E)** The wet/dry ratio of lungs from LPS-challenged mice treated with different formulations of Cur and RV. Data were expressed as mean ± SD (n = 4/group). *p < 0.05, **p < 0.001 *versus* vehicle group.

To confirm the effect of PM@Cur-RV NPs on targeting inflammatory lungs, an IVIS imaging system was performed. Indocyanine green (ICG) was co-loaded onto the nanoparticles as the fluorescent marker. The protocol and time-axis of *in vivo* targeting tests were shown in Figure 3A. Mice were intraperitoneal injected (*i.p.*) with LPS (8 mg/kg) to induce ALI. Fluorescent (ICG) mixed with free Cur-RV, Cur-RV NPs or PM@Cur-RV NPs were then *i. n.* Administered to visualize the tracks of NPs in the lungs. Figure 3 showed the distribution of NPs in the body at different time points after inhalation of NPs. Figure 3C showed that PM-coated biomimetic nanoparticles significantly promoted the accumulation and retention in inflammatory lungs. At 4 h of inhalation of NPs, mouse organs were collected for IVIS imaging. As shown in Figure 4D, all formulations of drugs were accumulated in the lungs but not in other organs, and the Cur-RV NPs, particularly PM@Cur-RV NPs, showed more stronger MFI in the lungs than other main organs. The decreased MFI of free ICG/Cur-RV could be due to the fast clearing out by the immune system. These results demonstrate that platelet membrane decoration of NPs has improved drug delivery and accumulation in inflammatory lungs.

### PM@Cur-RV NPs significantly reduce lung injury and vascular permeability

ALI is characterized by leukocyte accumulation, epithelial injury, pulmonary edema, and increased alveolar permeability, as well as diffuse alveolar damage. It usually leads to ARDS, and is the primary cause of death in critical patients (Butt et al., 2016). We explored the lung vascular permeability by determining the Evans blue albumin (EBA) flux (permeability to protein) (Nidavani RBM and Shalawadi, 2014; Wick et al., 2018). Figure 4A illustrated the experimental scheme of the lung vascular permeability assay. The mice were *i. p.* Administrated with LPS (5 mg/kg), and then different drugs were *i. n.* Administrated 3 h-post LPS challenge. At 23.5 h LPS challenge, the mice were intravascular injected with EBA. After 30 min, mouse lungs were collected to determine the EBA flux. Figure 4B showed the representative images of the lungs extracted from the EBA-injected-mice. As expected, LPS treatment resulted in increased EBA flux at 24 h post-LPS compared to the basal controls. Mice received the free Cur and RV treatment were no discernable improvements in lung permeability compared to the vehicle treatment. However, mice received Cur-RV NPs, especially PM@Cur-RV NPs, showed remarkably decreased EBA flux levels of lung vascular permeability (Figure 4C). These results demonstrate that nasally administered PM@Cur-RV NPs leads to targeted delivery of Cur and RV to inflammatory lungs and effectively inhibits pneumonia permeability. The protein levels in broncholveolr lvge fluid (BALF) and wet/dry ratio were also detected to evaluate the severity of pulmonary edema. Figures 4D,E demonstrated that NPs treatment was significantly attenuated the pulmonary edema in ALI mice.

### Anti-inflammatory efficacy of PM@Cur-RV NPs in LPS-Induced ALI mice

**FIGURE 5:**
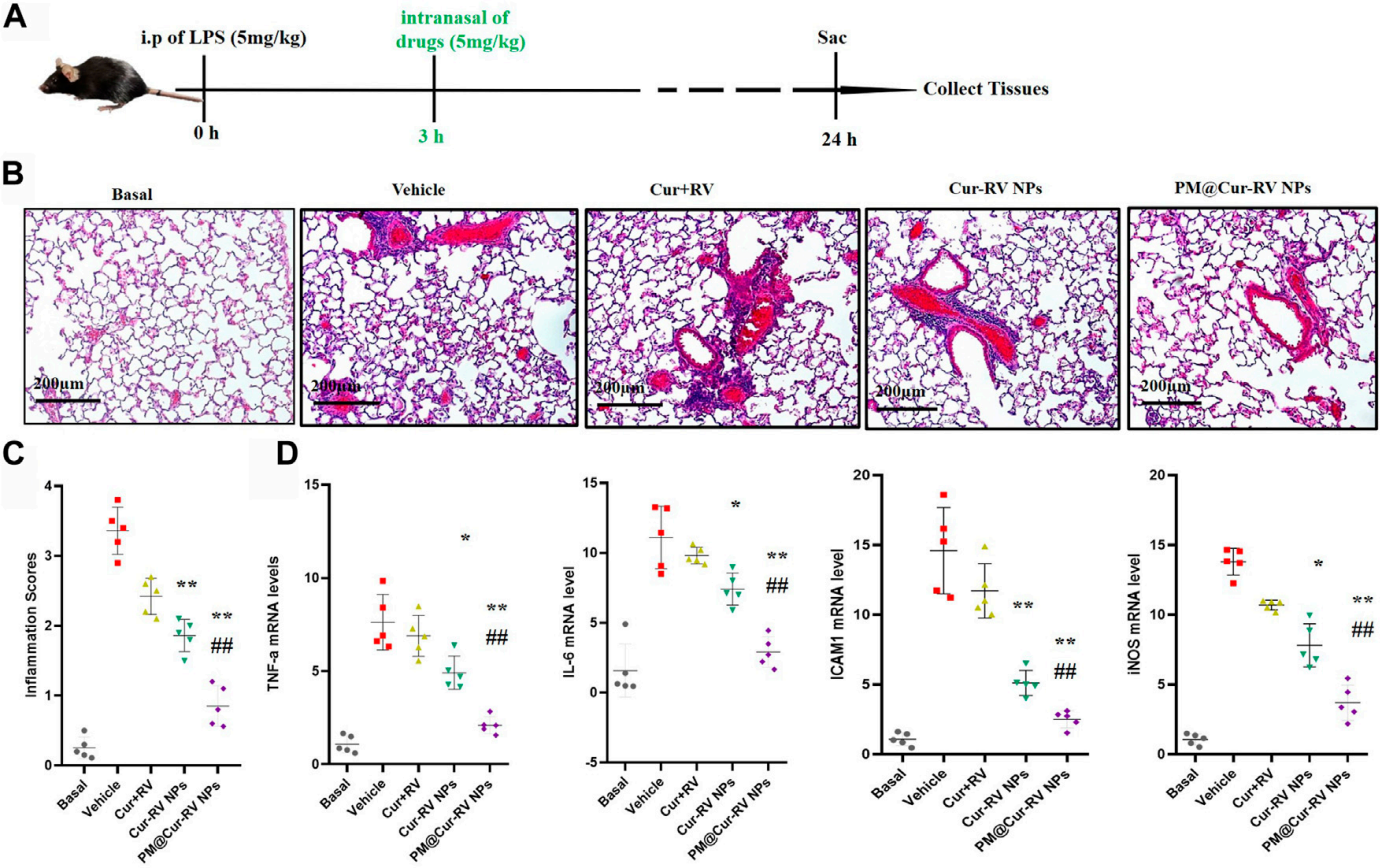
Cur-RV NPs, especially PM@Cur-RV NPs significantly promote resolution of lung inflammation in LPS-induced septic mice. **(A)** Time-axis of lung inflammation determination. The mice were intranasally administrated different formulations of Cur and RV at 3 h post-LPS challenge (5 mg/kg), and bronchiolar alveolar lavage fluid (BALF), blood, and lung tissues were collected at 24 h post-LPS challenge (5 mice/group). **(B)** Representative micrographs of lung tissue cross-sections at 24 h post-LPS challenge stained by H&E. Magnification (×400). **(C)** Inflammation scores in the different treated groups. **(D)** Expression levels of pro-inflammation cytokines TNF-a, IL-6, ICAM1 and iNOS, respectively. *p < 0.05, **p < 0.001 versus vehicle group.

The therapeutic effect of Cur and RV was examined in an LPS-induced ALI model. Mice were *i. p.* Injected with LPS at 5 mg/kg, and drugs were *i. n.* Administrated at 3 h-post LPS challenge. In Figures 5B,C, hematoxylin and eosin (H&E) staining showed that Cur-RV NPs, particularly PM@Cur-RV NPs-treated mice exhibited less alveolar wall thickening and inflammatory cell infiltration in the pulmonary alveoli and attenuated inflammatory cell recruited to lung tissues, indicating a protection effect of Cur-RV NPs against lung injury. Moreover, the proinflammation cytokines in ALI mice, such as TNFα, IL-6, ICAM1 and iNOS, were significantly increased (Figure 5D). However, all these cytokines were significantly inhibited after treated with Cur-RV NPs, especially PM@Cur-RV NPs. Collectively, PM@Cur-RV NPs show a higher efficacy therapeutic benefit in the ALI model by inhibiting the infiltration of inflammatory cell and relieving proinflammatory cytokines.

### Toxicological studies in ALI mice

**FIGURE 6:**
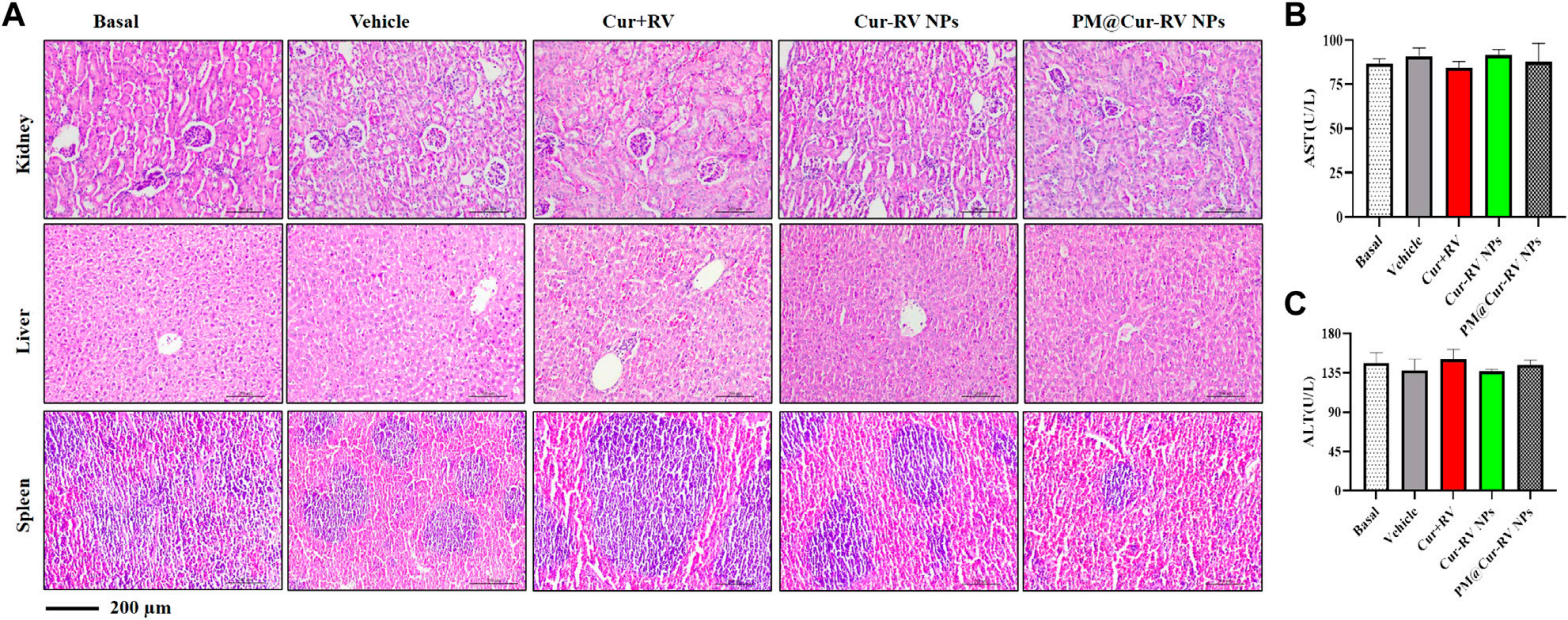
Toxicological studies in ALI mice. **(A)**Pathological data using H&E staining of different organs (Kidney, Liver and Spleen) originated from ALI mice inhaled with different compounds. No obvious toxicity was observed. Magnification (×400). **(B–C)** The levels of aspartate aminotransferase (AST) and alanine transam (ALT) of livers were tested to evaluate the toxicity of different formulations of Cur-RV. There was no statistical significance among the groups.

The biosafety of drugs is very important in their clinical applications. We analyzed the cytotoxicity of PM@Cur-RV NPs *in vivo* by histological analysis of the major organs (kidney, spleen, liver) (Huang et al., 2018b). H&E staining revealed that no significant tissue damage and adverse effect to these organs in all test groups (Figure 6A). We also explored the levels of aspartate aminotransferase (AST) and alanine transam (ALT) of livers to evaluate the effects of drugs to liver functions (Figure 6B) (Zhao et al., 2020). There was no statistical significance among all the treated groups of ALI mice. This result implies that PM@Cur-RV NPs possess excellent biocompatibility and biosafety, indicating a promising potential in clinical applications.

### PM@Cur-RV NPs induce macrophage polarization towards M2 in ALI mice

**FIGURE 7:**
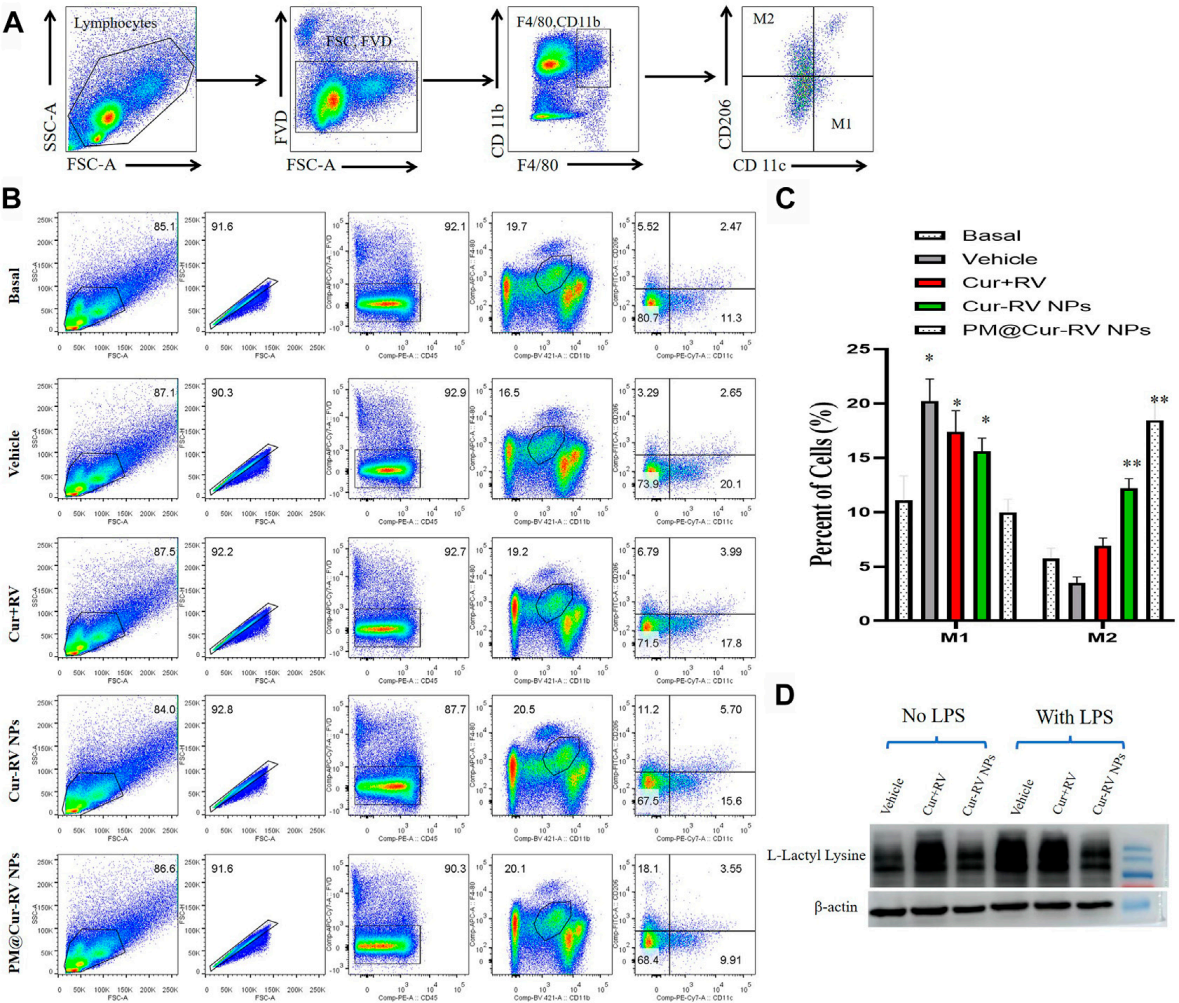
PM@Cur-RV NPs reduce lung inflammation by inducing macrophage towards to M2 polarization. Flow cytometric analysis of macrophages derived from inflammatory lungs treated with different formulations of Cur and RV for 24 h. **(A)** The scheme illustrates the selection of M1-or M2-positive cells from isolated single cells of lung tissues. In brief, we first gated out the live cells, and then gated out the F4/80-positive cells. In this subgroup, we used CD11c and CD206 as markers to identify M1 or M2 macrophages. F4/80+, CD11c+, CD206-cells are zoned as M1 cells, F4/80+, CD11c-, CD206+ cells are marked as M2 cells. **(B)** The representative images of cell distribution pattern in sepsis mice treated with different formulations of drugs. **(C)** The quantitative analysis of percentage of total M1 (F4/80+) macrophages and M2 (CD206+) macrophages in LPS-challenged lungs (n = 4/group; **p < 0.01, ***p < 0.0001 *versus* PBS-treated group; ^##^ *p* < 0.001, ^###^ *p* < 0.001 *versus* Nar-treated group). **(D)** Representative western blot of L-Lactyl Lysine expression in RAW264.7 cells after 24 h treatment with vehicle or different formulations of Cur-RV. β-actin was used as a loading control. It shows that Cur-RV significantly decreased the lactic acid modification in LPS-induced macrophages.

Activated macrophages are polarized into two categories: M1 (F4/80^+^CD11c^+^CD206^–^) macrophages, which are mainly associated with pro-inflammatory responses; and M2 (F4/80^+^CD11c^–^CD206^+^) macrophages, which are mainly associated with anti-inflammatory responses. Figure 6A demonstrated the method to analyze M1 and M2 cells in all isolated monocytes from lung tissues. As shown in Figure 7B, macrophages in the lung tended to polarize to M1 phenotype in LPS-challenged ALI mice. After treatment with Cur and RV, we found that all formulations of Cur and RV-treated group, especially PM@Cur-RV NPs, significantly decreased the number of M1 macrophages, and oppositely increased the number of M2 macrophages in the lungs (Figures 7B,C), suggesting that Cur and RV-treatment induces alveolar macrophages polarized from M1-type to M2-type.

Huge amount of studies indicate that elevated and impaired clearance of blood lactate are independently involved in increased mortality in septic patients (Marty et al., 2013). Thus, the use of lactate clearance as a treatment guideline for sepsis has gained increasing attractions in adult and pediatric patients in clinics (Marty et al., 2013). Figure 7D showed that Cur-RV treatment significantly decreased the levels of histone lactylation in LPS-induced macrophages. This indicates that Cur- RV attenuates ALI might be through the pathway of decreased histone lactylation and regulating macrophage to M2 polarization, however, further study need to be done in future study.

## Discussion

The respiratory system plays crucial roles in the biology of vertebrate animals. Injuries of the respiratory system caused by bacterial or viral infections (e.g., by COVID-19 and SARS) can lead to severe or lethal conditions. To date, there are no effective treatments for respiratory injuries. Recently, the COVID-19 pandemic caused a huge economic and medical burden to the whole world. Therefore, it is impressive to develop effective drugs and strategies to fight against infection-induced respiratory injuries. In the past decades, the rapid development of bio-nanotechnology provides new ideas for people to fight diseases. Nanomedicines are utilized to deliver a variety of therapeutic agents, including corticosteroids, chemotherapy drugs, herbal extract and nucleic acids (Blanco et al., 2015).

Actively targeting strategies aim at improving drug homing simultaneously reducing systemic toxicity (Bazak et al., 2015). Interestingly, cell membrane-cloaked biomimetic nano-formulations are capable of possessing the properties of the inherent tropisms of the host cells and the therapeutic function of drugs encapsulates in the nano-core (Tan et al., 2015). In this work, we develop a biomimetic nano-delivery system for localizing the therapeutic payloads to the lungs. Platelet cell vesicles, homing to the inflammation sites, are employed to coat nanoparticle cores loaded with the anti-inflammatory herbal ingredients-Cur and RV. The as-synthesized PM@Cur-RV NPs are used to treat ALI mice through inhaled administration.

In clinics, most of drugs are currently administrated intravenously. Topical administration of drugs to the disease sites highlights a better safety and efficacy. In the current study, the efficacy of inhaled PM@Cur-RV NPs demonstrates a high efficiency in anti-inflammation and attenuation of ALI. Comparing with an intraperitoneally administration of Cur at 200 mg/kg (Kim et al., 2016) or RV at 30 mg/kg (Li et al., 2013), the 20-fold lower dose administered by inhalation represents a comparable curative effect in ALI mice. This result supports further clinical development of inhalation nano-drugs against pulmonary disease.

Vascular permeability is characterized by the plasma and its solutes cross the vascular barrier. It is essential for the health of normal tissues and is also a vital factor in many disease states such as tumors, wounds, and inflammatory diseases. The acute inflammatory response quickly induces the increased vascular permeability, and leukocytosis into the injured tissues (Zhao et al., 2014). In the pathological processes of ALI, the increase in lung vessel permeability and loss of alveolar-capillary membrane integrity are closely associated with neutrophils infiltration and cytokine storm release in the lungs (Mammoto et al., 2013).

Recent studies demonstrate that high dose of Cur and RV can effectively inhibit the production of pro-inflammatory cytokines, however, their anti-inflammatory effects are greatly limited *in vivo* and must need high dose for that purpose (>100 mg/kg) (Li et al., 2013; Kim et al., 2016). Only the high dose could achieve the curative effects in experimental animal models, due to their poor water solubility, lower biocompatibility and bioavailability. Using nano-formulation, 5 mg/kg of PM@Cur-RV NPs can reach high treatment efficiency on ALI. Disruption of endothelial barrier leads to lung edema and injury, and PM@Cur-RV NPs might regulate endothelial barrier function and decrease vascular permeability in our study.

Macrophages in the lungs sustain the homeostasis *via* phagocytosing the inhaled particulate, foreigner pathogens and inducing cytokine production or antigen presentation, which facilitates the clearance of particulate antigens (Puttur et al., 2019). Macrophage polarization is a process in which macrophages phenotypically mount a specific phenotype and functional response under different pathophysiological conditions and microenvironments (Biswas et al., 2012). The balance of M1 and M2 macrophages could help to avoid excessive inflammatory responses which can lead to tissue damage. Macrophages are usually polarized to M1 phenotype in response to microbial stimuli including lipopolysaccharide (LPS) and Th1-related cytokines such as IFNγ and TNFα (Murray et al., 2014). M1 macrophages produce large amounts of inflammatory mediators and cytokines such as TNFα, IL-1, IL-6, IL-12, iNOS, reactive oxygen species (ROS), *etc.* These inflammatory mediators simultaneously promote inflammatory response and Th1 immune response and even cause tissue damage (Aggarwal et al., 2014). In the early stage of ALI/ARDS, alveolar macrophages are M1-polarized and release several pro-inflammatory factors, which facilitate to clear pathogenic microorganisms and recruit neutrophils. However, the excessive accumulation of pro-inflammatory cytokines and inflammatory cells reversely leads to lung tissue injury (Huang et al., 2018a). M2 macrophages contribute to promoting the recovery of host tissues through releasing anti-inflammatory mediators, inhibiting the production of proinflammatory cytokines, and removing apoptotic neutrophils from the inflammatory sites (Wynn and Vannella, 2016). Thus, the regulation of macrophage polarization from M1 towards M2 macrophages is the key regulator of tissue repair during ALI/ARDS recovery.

Moreover, our study have reported that lactate is elevated in ALI, and it might influence the function of immune cells (Caslin et al., 2021). Lactic acid-mediated glycolytic inhibition may suppress the function of inflammatory immune cells and promote their regulatory function (Nolt et al., 2018). It has been reported that the acute hospital mortality is significantly higher in patients with higher serum lactate level than those with lower serum lactate level (Lee and An, 2016). Decreased or normalized the lactate levels are an important sign of recovery from septic shock, and lactate clearance at a discrete time point is an important prognostic factor compared to the initial serum lactate level in severe sepsis (Lee et al., 2015). In this study, we have found that Cur-RV especially PM@Cur-RV NPs treatment significantly decrease the lactylation modification in macrophages and might regulate them into M2 phenotype. These results demonstrate a potential application of PM@Cur-RV NPs for fighting against inflammatory pulmonary disease, such as ALI.

## Conclusion

In this study, we developed a platelet membrane-cloaked nanoplatform to deliver anti-inflammation drugs for ALI treatment. The as-synthesized PM@Cur-RV NPs exhibited excellent dispersion, good biocompatibility and increased biosafety *in vitro* and *in vivo*. This biomimetic nanoplatform could successfully target and accumulate into inflammatory lungs in ALI mice. In LPS-induced ALI mice, the PM@Cur-RV NPs significantly improved the anti-inflammatory efficiency, suppressed the vascular permeability of lung edema, and decreased the inflammatory cells infiltration, which demonstrated an excellent enhanced therapeutic efficiency comparing with the free Cur-RV. Furthermore, flow cytometry analysis indicated that PM@Cur-RV NPs polarized lung macrophages from M1 to M2 type. Notably, the effective dose of Cur or RV in this work (1 mg/kg) was decreased more than 20-fold comparing with their effective doses reported in the previous studies (10 mg/kg for inhalation (Kumari et al., 2015) and 100 mg/kg for intraperitoneal injection (Guzel et al., 2013), which highlighted the merits of nanoplatform-based system in drug delivery to disease location. Together, this work provides a promising biomimetic nanoplatform for targeting treatment of inflammatory pulmonary disease, and this strategy shows potential clinical application in the future.

## Data Availability

The original contributions presented in the study are included in the article/supplementary material, further inquiries can be directed to the corresponding authors.
